# Ammonia Reduces Intracellular Asymmetric Dimethylarginine in Cultured Astrocytes Stimulating Its y^+^LAT2 Carrier-Mediated Loss

**DOI:** 10.3390/ijms18112308

**Published:** 2017-11-02

**Authors:** Krzysztof Milewski, Małgorzata Bogacińska-Karaś, Inez Fręśko, Wojciech Hilgier, Radosław Jaźwiec, Jan Albrecht, Magdalena Zielińska

**Affiliations:** 1 Department of Neurotoxicology, Mossakowski Medical Research Centre, Polish Academy of Sciences, 02-106 Warsaw, Poland; kmilewski@imdik.pan.pl (K.M.); mkaras@imdik.pan.pl (M.B.-K.); inezfresko@gmail.com (I.F.); whilgier@imdik.pan.pl (W.H.); jalbrecht@imdik.pan.pl (J.A.); 2 Mass Spectrometry Laboratory, Institute of Biochemistry and Biophysics, Polish Academy of Sciences, 02-106 Warsaw, Poland; rjazwiec@gmail.com

**Keywords:** astrocytes, rat brain endothelial cell line (RBE-4) cells, ammonia, nitric oxide, asymmetric dimethylarginine (ADMA), symmetric dimethylarginine (SDMA), y^+^LAT2

## Abstract

Previously we had shown that ammonia stimulates nitric oxide (NO) synthesis in astrocytes by increasing the uptake of the precursor amino acid, arginine via the heteromeric arginine/glutamine transporter y^+^LAT2. Ammonia also increases the concentration in the brain of the endogenous inhibitor of nitric oxide synthases (NOS), asymmetric dimethylarginine (ADMA), but distribution of ADMA surplus between the intraastrocytic and extracellular compartments of the brain has not been studied. Here we tested the hypothesis that ammonia modulates the distribution of ADMA and its analog symmetric dimethylarginine (SDMA) between the two compartments of the brain by competition with arginine for the y^+^LAT2 transporter. In extension of the hypothesis we analyzed the ADMA/Arg interaction in endothelial cells forming the blood-brain barrier. We measured by high-performance liquid chromatography (HPLC) and mass spectrometry (MS) technique the concentration of arginine, ADMA and SDMA in cultured cortical astrocytes and in a rat brain endothelial cell line (RBE-4) treated with ammonia and the effect of silencing the expression of a gene coding y^+^LAT2. We also tested the expression of ADMA metabolism enzymes: protein arginine methyltransferase (PRMT) and dimethylarginine dimethyl aminohydrolase (DDAH) and arginine uptake to astrocytes. Treatment for 48 h with 5 mM ammonia led to an almost 50% reduction of ADMA and SDMA concentration in both cell types, and the effect in astrocytes was substantially attenuated by silencing of the *Slc7a6* gene. Moreover, the y^+^LAT2-dependent component of ammonia-evoked arginine uptake in astrocytes was reduced in the presence of ADMA in the medium. Our results suggest that increased ADMA efflux mediated by upregulated y^+^LAT2 may be a mechanism by which ammonia interferes with intra-astrocytic (and possibly intra-endothelial cell) ADMA content and subsequently, NO synthesis in both cell types.

## 1. Introduction

Hyperammonemia (HA) is defined as a clinical condition in which the ammonium ion concentration in the blood and brain exceeds 130 and 500 µM respectively [1,2]. HA contributes to progression of neurological disorders including acute and chronic forms of hepatic encephalopathy, but also to congenital deficiencies of urea cycle enzymes, Reye’s syndrome, and several metabolic or toxic encephalopathies [3]. One of the principle mechanisms by which ammonia damages the brain is oxidative/nitrosative stress (ONS) [4]. ONS is a result of enhanced production of reactive oxygen species (ROS) followed by rapid reaction with NO to produce highly reactive nitrogen species (RNS) [5]. Activation of NO synthesis by ammonia contributes to ONS in astrocytes [6,7,8], neurons [9], and endothelial cells (e.g., rat brain endothelial cell line (RBE-4) cell line) alike [10]. While in neurons induction of NO synthesis by ammonia is a straight consequence of over-activation of neuronal NMDA receptors [11], in astrocytes it is prompted by increased uptake of the NO precursor arginine [12,13]. This process is facilitated by increased expression of the heteromeric glutamine/arginine transporter y^+^LAT2 as demonstrated in ammonia treated astrocytes and RBE-4 cells [14,15], but also in the brain cortex of HA rats [14].

**Scheme 1:**
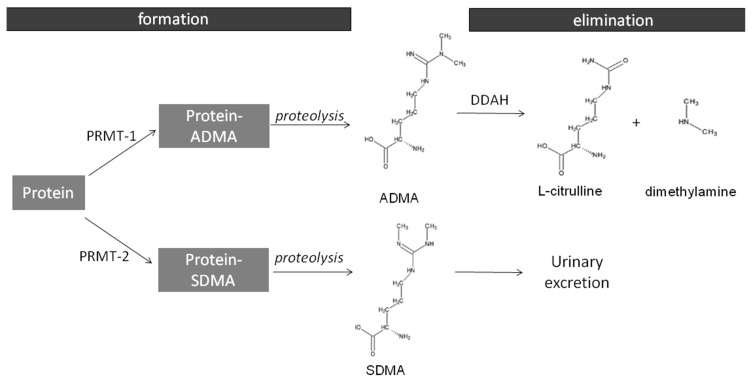
Asymmetric dimethylarginine (ADMA) and symmetric dimethylarginine (SDMA) are formed by the methylation of arginine residues in a variety of proteins catalyzed by protein arginine methyltransferases (PRMT). After proteolysis, arginine and its methylated forms are released as free forms. ADMA elimination occurs primarily and specifically by its degradation by dimethylarginine dimethyl aminohydrolases (DDAH-1 and -2), wherein only DDAH-1 is widely expressed in the brain [20,21]. In turn, SDMA is extracted in urine.

Asymmetric dimethylarginine N^G^, N^G^-dimethyl-l-arginine (ADMA), is the most abundant methylated arginine derivative in mammalian cells [16]. Unlike its analog N^G^, N^G’^-dimethyl-l-arginine (symmetric dimethylarginine; SDMA), ADMA inhibits NO synthesis by competing with arginine at the active site of nitric oxide synthase (NOS; EC 1.14.13.39) [17]. Furthermore, even relatively small changes in intracellular l-Arginine/ADMA ratio can affect NOS activity in vivo and in vitro [18,19]. Metabolism of dimethylarginines are outlined on Scheme 1.

ADMA can compete with arginine for two major cationic amino acid membrane transporters belonging to two related protein families: cationic amino acid transporters (system y^+^) and the system y^+^L, the latter represented by 4F2hc/y^+^LAT1 and 4F2hc/y^+^LAT2 [22]. HA is associated with increased ADMA concentration in the brain tissue [23] and brain extracellular fluid [24], but the mechanisms regulating distribution of the ADMA/SDMA surplus between the particular intra and extracellular compartments have not been studied. We hypothesized that treatment of astrocytes with ammonia may elicit ADMA export from the cells by y^+^LAT2, a process likely to be facilitated by upregulation of the carrier and competition by increased arginine uptake observed under these conditions [13]. In this study this hypothesis was tested in cultured astrocytes. Rat brain endothelial cells were considered as one other brain compartment in which the mechanism operates.

## 2. Results

### 2.1. The Effect of Ammonia and Slc7a6 Silencing on ADMA and SDMA Intra/Extracellular Concentration in Cultured Astrocytes

**Figure 1:**
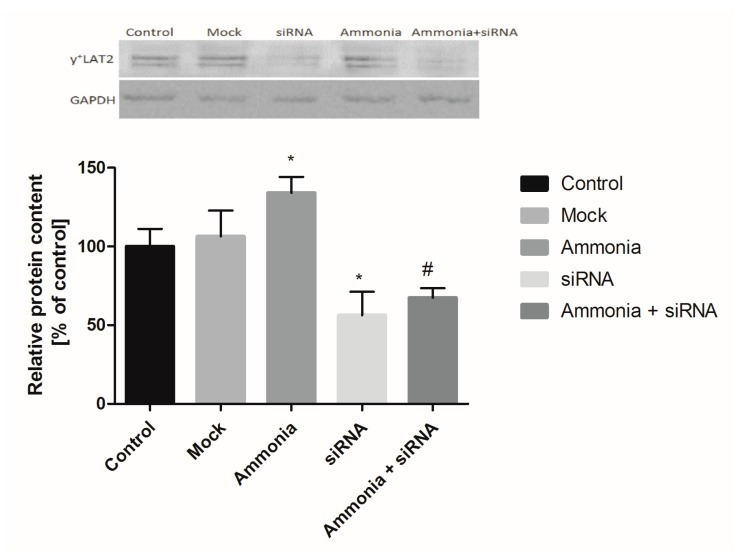
The effect of silencing of the *Slc7a6* gene on y^+^LAT2 protein level in astrocytes in relation to glyceraldehyde-3-phosphate dehydrogenase (GAPDH) protein level. Upper panel shows representative Western blots. Results are mean ± SD; *n* = 4, * *p* < 0.05 vs. control; ^#^ *p* < 0.05 vs. ammonia.

The efficiency of *Slc7a6* gene silencing procedure was tested first. A 48 h treatment with *Slc7a6* siRNA reduced y^+^LAT2 protein level by ~50%. Sequences of siRNA complementary to y^+^LAT2 mRNA completely abolished ammonia-induced increase of y^+^LAT2 expression (Figure 1).

The effect of ammonia treatment and *Slc7a6* gene silencing in primary cultured astrocytes obtained by high-performance liquid chromatography (HPLC) is shown in Table 1. Incubation with 5 mM ammonia reduced both ADMA (by ~38%) and SDMA (by ~44%) without significantly changing Arg level. Silencing of *Slc7a6* gene prevented ammonia-induced intracellular ADMA and SDMA reduction in astrocytes, indicating that y^+^LAT2 mediates transmembrane transport of dimethylarginines in these cells.

Our hypothesis was supported by mass spectroscopic analysis of dimethylarginines in culture medium (Table 2). Ammonia increased extracellular ADMA and SDMA concentration by 15% and 39% respectively. This effect was abolished by *Slc7a6* gene silencing. Freshly prepared medium does not contain detectable amounts of dimethylarginines as was verified using the procedure included in Materials and Methods section.

To test if the effects of ammonia also comprise brain endothelial cells we measured intracellular ADMA and SDMA concentration in rat brain endothelium cell line—RBE-4. Ammonia caused almost 50% reduction in ADMA and SDMA level in RBE-4 cells (Table 3).

The concentration of ADMA and SDMA measured in control untreated RBE-4 cells was markedly higher than in astrocytes (Table 1 and Table 3, respectively). Astrocytes were characterized by a higher intracellular l-arginine/ADMA ratio (~25) than RBE-4 cells (~11). The central role of astrocytes in ammonia neurotoxicity prompted us to focus further experiments within this study on astroglial cells.

### 2.2. The Expression of ADMA-Metabolizing Enzymes

**Figure 2:**
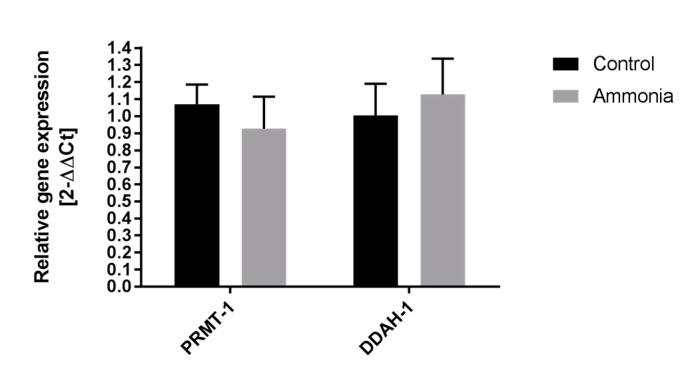
Expression of mRNAs coding for enzymes of ADMA metabolism, *PRMT1* and *DDAH1* genes in control and ammonia-treated astrocytes.

**Figure 3:**
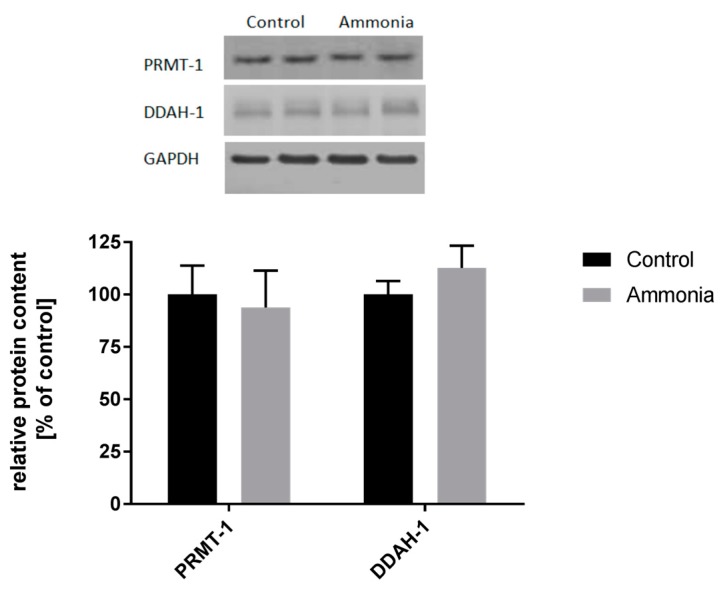
PRMT-1 and DDAH-1 protein level in primary astrocytes treated with 5 mM ammonia for 48 h. Upper panel shows representative Western blots.

As mentioned in the Introduction ADMA and SDMA each, use different enzymes for their synthesis and utilization. Identity of the patterns of ammonia-induced changes in ADMA and SDMA (Section 2.1) suggested that events other than alterations in their metabolism (for instances transport) are a more likely cause of reduction of intracellular ADMA concentration after ammonia treatment. Nonetheless to test this inference, we measured the gene expression and protein level of ADMA synthesizing enzyme PRMT-1 and a major utilization enzyme DDAH-1. Indeed, neither PRMT-1 nor DDAH-1 was altered after ammonia treatment (Figure 2 and Figure 3).

### 2.3. The Nitrite/Nitrate Production in Cultured Astrocytes

**Figure 4:**
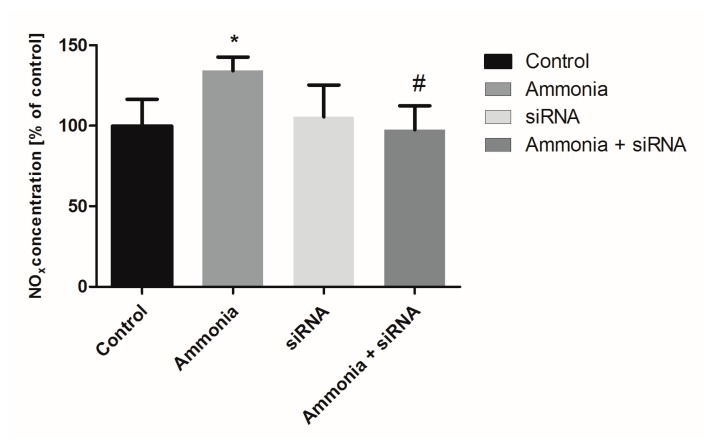
The effect of ammonia and *Slc7a6* gene silencing on nitrite+nitrate (NOx) concentration in the medium derived from cultured astrocytes. Result are mean ± SD; *n* = 5, * *p* < 0.05 vs control, ^#^ *p* < 0.05 vs. ammonia.

To determine whether observed depletion of intracellular ADMA and SDMA in ammonia treated cells may affect NO production we measured nitrites + nitrates (NO_x_) amount in the cultured medium. Ammonia enhanced by ~35% NO_x_ concentration in cultured astrocytes medium. The effect was attenuated in cells in which y^+^LAT2 protein was down regulate (Figure 4).

### 2.4. The Effect of ADMA on [^3^H]Arginine Uptake to Cultured Astrocytes

**Figure 5:**
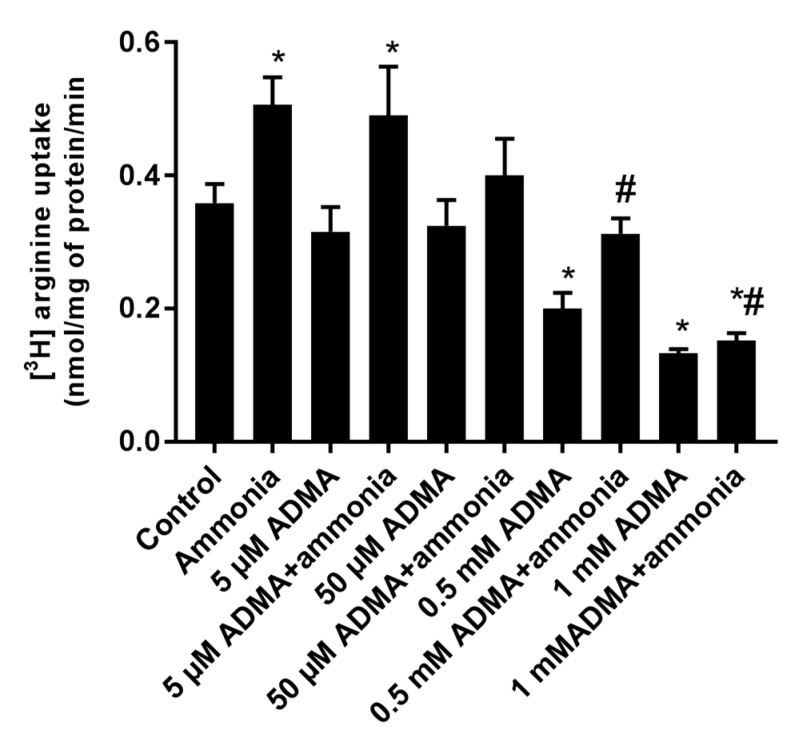
The effect of ADMA on [^3^H]arginine uptake in control and 5 mM ammonia treated primary astrocytes cultures. Result are mean ± SD; *n* = 6, * *p* < 0.01vs control, ^#^ *p* < 0.01 vs. ammonia.

ADMA may also act as inhibitor of arginine transport which inter alia is catalyzed by the y^+^L system. Incubation with 5 and 50 µM ADMA did not affect [^3^H]arginine uptake to astrocytes (Figure 5). Inhibition of [^3^H]arginine uptake was observed only at, relatively high, non-physiological ADMA concentrations (0.5 and 1 mM) (Figure 5).

## 3. Discussion

Excessive intracellular accumulation of reactive oxygen and nitrogen species (ROS and RNS, respectively) resulting in ONS induction in the brain is a hallmark of ammonia neurotoxicity [25]. Accumulation of ROS/RNS has been successfully documented in hyperammonemic conditions in situ, and in cultured astrocytes [8,26,27]. A distinct feature of the chain of reactions which translate ammonia overexposure to NO synthesis in astrocytes is increased uptake of the NO precursor arginine, a process facilitated by increased expression/activity of the heteromeric glutamine/arginine exchanger y^+^LAT2 [13,15]. Accordingly, down-regulation of the y^+^LAT2 carrier has been documented as a successful tool for the inhibition of NO synthesis and of the ensuing excessive S-nitrosylation in astrocytes [15].

The asymmetric dimethylarginine (ADMA) serves as endogenous inhibitor of NOS, modulating NO synthesis in various tissues [28,29,30]. Changes in the ADMA levels or l-arginine/ADMA ratio have been noted in plasma of patients with acute liver failure [31], or with liver cirrhosis [32]. Increased ADMA concentration in plasma and brain was also reported in animals with surgically or chemically induced HE [33,34]. Recent studies in our laboratory demonstrated that ADMA accumulates in the brain of rats with liver failure induced by thioacetamide [23,24]. However, the magnitude and mechanistic details of interference by ADMA with NO synthesis under hyperammonaemic conditions was not addressed by the previous studies and needs detailed elucidation.

A hypothesis has been put forward that ammonia-induced changes in ADMA content or distribution in the brain may modulate NO synthesis not only by straightforward interaction with NOS, but also by interfering with arginine availability for NO synthesis. This idea was prompted by data indicating competition of ADMA and arginine for cell membrane transport sites. The present study positively verified this hypothesis for astrocytes, the cells in which arginine overflow plays a critical role in ammonia-induced NO synthesis (see Introduction). The principal findings favoring the hypothesis are that (i) ammonia reduced intracellular ADMA concentration measured in astrocytes; (ii) silencing of y^+^LAT2 coding gene was associated with an increase of ADMA in the cells and decrease of NO production; (iii) the absence of the effects of ammonia on the protein levels of ADMA-metabolizing enzymes and the reduction of intracellular level of SDMA, arginine derivative metabolized via distinct enzymatic pathway (see Scheme 1) excludes classical, metabolic mechanism of ammonia action on ADMA content in astrocytes.

The present study extended our knowledge of described our previous study demonstrated that ammonia-induced increase of NO synthesis is associated with increased [^3^H]arginine uptake via upregulated in y^+^LAT2 [15]. The present study pointed to cellular ADMA depletion as one other potential cause of ammonia-induced NO increase which is coupled to upregulation of y^+^LAT2. In so far, this conclusion was derived from analysis of steady state ADMAi/ADMAe ratios. Clearly this conclusion will have to be verified by comparing ADMA transport kinetics in y^+^LAT2-rich and y^+^LAT2-deplete preparations.

Enhanced NO synthesis in astrocytes treated with ammonia is predominantly a result of inducible nitric oxide (iNOS) induction [15]. The presence of sound evidence of y^+^LAT2-iNOS coupling in ammonia-treated cultured astrocytes (15) did not a priori exclude the involvement endothelial nitric oxide synthase (eNOS) in modulation of NO synthesis by ammonia [35]. However, in contrast to iNOS, effective reduction of y^+^LAT2 transporter content did not affect eNOS mRNA expression, suggesting the absence of y^+^LAT2–eNOS coupling in astrocytes [15]. At this point it is worth noting that ADMA counteracts NO over-production caused by iNOS activation [36]. The possibility that ADMA inhibits other NOS isoforms as well is worth a separate investigation.

The present data suggest that a mechanism similar to astrocytes holds for the RBE-4 cells, a rat brain endothelial cell line mimicking endothelial cells forming the blood-brain barrier (BBB). Of note in this context, the induction by ammonia of transcellular passage of low molecular BBB markers in RBE-4 cells is associated with increased y^+^LAT2 expression. Down the same valley, inflammatory factors which are induced in hyperammonemic conditions, may activate iNOS in brain endothelial cells [37]. This could subsequently lead to y^+^LAT2-iNOS coupling. More detailed analysis of ADMA-mediated regulation of NO synthesis in brain endothelial cells are under way.

Intracellularly formed dimethylarginines are exported to the extracellular fluid and plasma. ADMA transport is mediated predominantly by cationic amino acid transporters (CATs) [18,37]. Recent in vitro study on human embryonic kidney cells suggested the involvement in ADMA redistribution of other transporters, such as organic cation transporter 2 and multidrug and toxin extrusion protein 1 [38]. Of note, in human embryonic kidney cells it is the y^+^LAT1 transporter which plays an important role in depletion of intracellular ADMA, purportedly leading to eNOS uncoupling [39]. The present study implicated, to our knowledge for the first time, functioning of the y^+^LAT2 transporter in the same capacity in ammonia-affected astrocytes. Worth mentioning in this context are substantial changes of transporter expression and activity in the whole brain of rats in HA associated with thioacetamide-induced acute liver failure [14]. Since the mechanisms by which the endogenous methylarginines are distributed between the extracellular and intracellular compartments are not well characterized, existing evidence documented the involvement of y^+^ cation transporters [40] that may have lower affinity but high capacity for ADMA [38]. However, our previous experiments documented a lack of y^+^ system activation in ammonia-treated astrocytes, countering the involvement of y^+^ cation transporters in the ammonia-induced enhancement of astrocytic NO synthesis [13].

Several groups have pointed out that elevation in ADMA (plasma and/or brain) concentration observed in HE patients and animal models result from impaired ADMA metabolism [28,30,32]. However, our present work showed that in primary astrocytes, y^+^LAT2 activity may modulate intra/extracellular ADMA distribution independently from activation of ADMA metabolism. This may be concluded from both unchanged expression and protein level of PRMT-1 and DDAH-1 in ammonia treated astrocytes and reduction of intracellular SDMA, a dimethylarginine, which is not metabolized via the same enzymatic pathway as ADMA (see Scheme 1). The possibility that enzyme activities pertinent to ADMA metabolism are altered by ammonia treatment can’t be excluded and definitely deserves further examination. Nevertheless, neither elevated ADMA synthesis nor lowered ADMA utilization in astrocytes can simply and directly explain the impact of *Slc7a6* silencing on ADMA intra/extracellular concentration. Therefore, we consider our observation of y^+^LAT2-dependent modulation of ADMA distribution a valid addition to the mechanism irrespective of the effect on the activities of enzymes. To support this statement, it’s worth noticing that decrease in DDAH activity was observed in the brain cortex of rats with thioacetamide-induced liver failure but not in the brain cortex of rats with hyperammonemia as have been demonstrated previously [21,41]. An additional point related to DDAH activity needs clarification. Oxidative stress, one of the manifestations of ammonia toxicity, was reported as a factor reducing DDAH activity in vitro. However even in the cited study lowering of DDAH1/2 protein was also documented [42]. Therefore, the impact of the decrease of DDAH activity on the response of NO synthesis to ammonia does not appear decisive. The overall picture may be even more complex. For instance, depression of DDAH1 expression or activity may be a result of polymorphisms of the *DDAH1* gene [43,44]; reduced DDAH1 transcript expression [21], or post-translational modifications such as oxidation of DDAH1 protein: these two possibilities were not addressed in this study.

In conclusion, this study provides evidence that y^+^LAT2 transporter may control ADMA elimination in a manner which offers protection against dysregulation of NO synthesis in ammonia-treated astrocytes, and most likely in rat brain endothelial cells in vitro. The relevance of the findings for understanding the role of changes in the ADMA distribution/content in the brain affected by hyperammonemia remains to be documented. We are also fully aware that conclusions from the present study need verification in the in vivo setting. Nonetheless, we considered classical competition experiments with ADMA in the mmolar range as a prerequisite of addressing our hypothesis. In so far, recent observation of increased extracellular ADMA content in the brain of rats with thioacetamide-induced HE [24] appears to support the possibility of ADMA loss from astrocytes and/or endothelial cells. Taken together this finding justifies further investigations on the role of ADMA as a modulator of NO production in the pathogenesis of hyperammonemic encephalopathies including HE.

## 4. Materials and Methods

### 4.1. Chemicals

ADMA, SDMA, ADMA-D6, ammonium chloride, were provided by the Sigma-Aldrich (St. Louis, MO, USA); HPLC grade ethanol (EtOH), HPLC grade formic acid, gradient grade acetonitrile (ACN) were purchased from Witko (Witko, Lodz, Poland). MQ Water was purified with Millipore (Millipore, Bedford, MA, USA).

### 4.2. Cell Culture and Treatments

Primary astrocyte cultures were prepared from cortices of newborn Wistar rats using the method described previously [13]. Briefly, cerebral cortex was isolated on ice, then passed through Nitex nylon netting (pore size 80 µm) into Dulbecco’s modified Eagle’s medium (DMEM; Gibco, Waltham, MA, USA) containing 20% fetal bovine serum (Gibco). Cells were grown in 37 °C in humidified atmosphere of 95% air and 5% CO_2_. Experiments were performed on 3-week astrocytes. RBE-4 cells were cultured on the MEM/Ham’s F10 medium (Gibco) with addition of fibroblast growth factor-basic bFGF (Gibco). Cells cultured for 4 days cells were used to further experiments. Astrocytes and RBE-4 cells were treated with 5 mM ammonium chloride (“ammonia”) which was added into cell culture medium for 48 h (1 M stock solutions of ammonium chloride were stored at −20 °C and added at indicated concentration to culture medium).

### 4.3. Silencing of the Gene Coding for y^+^LAT2 Transporter

To down-regulate the y^+^LAT2 transporter, transfection with a mix of four types of siRNA duplexes, consisting of 21 nucleotides was performed. Each type of siRNA was targeted to different gene region to obtain the most effective gene silencing. Three-week astrocytes were washed with PBS solution, trypsinized to detach cells and then seeded at a density of 0.7 × 10^5^ per well in 24-well culture plates in 0.4 mL of astrocytic growth medium. On the same day, cells were transfected with 5 nM of siRNA and 3 µL of HiPerFect transfection reagent (Qiagen Benelux BV, Venlo, the Netherlands)/per well, according to fast-forward protocol, designed for adherent cells and provided by manufacturer (Qiagen). The mock control (transfection procedure without addition of siRNA) were used to indicate whether the transfection process results in cytotoxicity or other non-specific effects. Cells were cultivated with the transfection complexes under normal growth conditions for 48 h and then used for further experiments.

### 4.4. Dimethylarginines and Arginine Determination in Cell Homogenates and Culture Media

For determination of cellular ADMA, SDMA and arginine content, cells were washed with PBS and homogenized by sonication in 5-sulfosalicylic acid and centrifuged at 12,000× *g* for 15 min. Supernatant was neutralized and lyophilized. The lyophilizate was resolved in 40 mM acetate buffer and loaded onto a column packed with AG50W-X8, previously conditioned with 20 mM acetate buffer. Interfering compounds, i.e., proteins, acidic and neutral amino acids and weakly basic amines, were rinsed off the cartridge with 3 mL of 20 mM acetate buffer (pH 5.5), followed by 4 mL of water, 2 mL of 0.05 M ammonia, 8 mL of water, and 1 mL of methanol. Arg and its methylated metabolite were eluted with 3 mL of 25% aqueous ammonia-methanol (1:1, *v*/*v*) [45].

Arginine, ADMA and SDMA concentration were analyzed using HPLC with fluorescence detection after derivatization in a timed reaction with o-*phthal* aldehyde plus mercapto-ethanol, as described earlier [23].

The levels of ADMA, SDMA and arginine in cell culture medium were analyzed using positive mode electrospray LC–DMS–MS/MS. The deproteinized cell culture medium was mixed with ADMA-D6 (IS) solution in EtOH (4 ng/mL). Solution was evaporated to dryness under nitrogen, reconstituted with 45 µL of acetonitrile, and transferred to chromatographic vials. Samples were analyzed using Waters Xevo TQ-S triple quadrupole mass spectrometer coupled with Waters Acquity I-Class UPLC. A 3-min HPLC method was set up on Waters HILIC equipped with 1.7 µm 2.1 × 100 mm column with thermostatic control at 70 °C. Mobile phase A was composed of 0.1% FA in ACN, mobile phase B 0.1% FA in MQ. Linear gradient from 20% to 70% of phase B was used within 2.1 min with the flow rate of 0.65 mL/min. Injection volume was 3 μL. MS detector worked in ESI ionization in MRM mode. Separation was achieved by setting up MRM transitions that were highly specific for each compound as it was previously reported in literature [46].

### 4.5. l-[^3^H]Arginine Uptake Assay

l-[^3^H]Arginine uptake was measured in control or y^+^LAT2 silenced astrocytes, treated or not with 5 mM ammonium chloride. Cells were washed three times with Krebs buffer (Sigma-Aldrich, St. Louis, MO, USA). Basal arginine concentration in the cells was adjusted to 100 μmolar concentration by incubation in Krebs buffer containing 100 μM arginine for 10 min in 37 °C. Thereafter the solution was replaced by incubation mixtures containing Krebs buffer with 0.1 μCi/mL l-[^3^H]Arginine (specific radioactivity 37 MBq/mL; Hartmann Analytic) unlabeled arginine at 100 μmol/L and ADMA in the concentrations: 5 μM, 50 μM, 0.5 mM, 1 mM, and the incubation was continued for 20 min. Cells were then washed three times in ice-cold Krebs buffer, lysed in 0.5 mL 1M NaOH and protein content was determined. The radioactivity of cell lysates was measured in a Wallac 1409 (Perkin–Elmer, Turku, Finland) liquid scintillation counter.

### 4.6. Real-Time PCR Analysis

Total RNA from astrocytes was isolated using TRI Reagent (Sigma-Aldrich), and 1 μg of the RNA was reverse transcribed using the high-capacity cDNA reverse transcriptase kit (Applied Biosystems, Waltham, MA, USA). Real-time PCR was performed in 96-well plates with the ABI 7500 apparatus (Applied Biosystems) using the Applied Biosystems Taqman probe assays: PRMT-1 (Rn 00821202), DDAH-1 (Rn 00574200) β-actin (Rn 00667869). Each reaction contained 5 μL Taqman Universal PCR Master mix in a total volume of 10 µL, and 1 μL cDNA was added to the reaction. The real-time PCR reactions were performed for 10 min at 95 °C, followed by 40 cycles of 15 s at 95 °C, and 1 min at 60 °C. The results of the analysis were calculated in relation to the β-actin product, and results were presented according to an equation (2^−ΔΔ*C*t^) that gives the amount of target, normalized to an endogenous reference, and relative to a calibrator. *C*_t_ is the threshold cycle for target amplification [47].

### 4.7. Protein Isolation and Western Blot Analysis

Astrocytes were washed in PBS and homogenized by sonication in RIPA Lysis and Extraction buffer (Sigma-Aldrich) containing Protease Inhibitor Cocktail (concentration 1:200, Sigma-Aldrich) phosphatase inhibitor cocktail (concentration 1:100, Sigma-Aldrich) andf 50 μM sodium fluoride 0.5 M (Fluka, Sigma-Aldrich) and then centrifuged for 10 min at 10 000× *g* and 4°C. The supernatants was transferred to a new Eppendorf tube. Equal amounts of protein (30 μg) were separated on 10% SDS-polyacrylamide gel and transferred onto nitrocellulose membrane. Membranes were blocked with 5% nonfat dry milk in TBS-Tween buffer (50 mM Tris; 150 mM NaCl; 0.1% Tween 20). The membranes were than incubated over-night with anti-y^+^LAT2 antibody (1:1000, Sigma-Aldrich), anti-PRMT-1 (1:1000 Cell Signaling, Danvers, MO, USA) and anti-DDAH-1 (1:250 Santa Cruz Biotechnologies, Dallas, TX, USA), followed by 1 h incubation with HRP-conjugated-secondary IgG antibodies (1:5000, Sigma-Aldrich) for detection by Clarity Western ECL Substrate (Bio-Rad Laboratories, Hercules, CA, USA). The first antibody was stripped off with 0.1 M glycine, pH 2.9, and second incubation was performed with an antibody against Glyceraldehyde 3-phosphate dehydrogenase (GAPDH) 1 h incubation at 20–22 °C (1:7500, HRP-60004, ProteinTech, Manchester, UK). The chemiluminescent signal acquisition and densitometry analysis were conducted using the G-Box system (SynGene, Bengaluru, India) and GeneTools software (SynGene).

### 4.8. Determination of Nitric Oxide (NO) Concentration

As an index of NO production, nitrites amount in the cultured medium were measured together with nitrates (nitrite + nitrate = NO_x_) using Nitrate/Nitrite Colorimetric Assay Kit (Cayman Chemical Company, Ann Arbor, MI, USA), according to the manufacturer’s protocols.

### 4.9. Protein Determination

Total protein concentration was determined by the Lowry method using Modified Lowry Protein Assay Reagent (Pierce, Thermo Fisher, Waltham, MA, USA).

### 4.10. Statistical Analysis

Biochemical parameters were analyzed by one-way ANOVA with a post hoc Dunnet’s test. All analyses were performed using 5.0 Graph Pad software (San Diego, California, USA). Values were expressed as mean ± S.D.; *p* < 0.05 or less was considered statistically significant.

## Figures and Tables

**Table 1:** Intracellular ADMA/SDMA/arginine (Arg) concentrations in ammonia-treated astrocytes and effect of *Slc7a6* gene silencing. Results are mean ± SD; *n* = 8 (control; ammonia) *n* = 4 (Mock control; ammonia + y^+^LAT2 siRNA); * *p* < 0.05 vs. respective control; ^#^ *p* < 0.05 vs. ammonia.

Intracellular Concentration	Control	Ammonia	Mock Control	Ammonia + y^+^LAT2 siRNA
ADMA (µM)	7.3 ± 1.01	4.38 ± 1.80 *	6.91 ± 1.47	8.29 ± 0.84 ^#^
SDMA (µM)	5.82 ± 1.32	3.27 ± 1.18 *	5.29 ± 1.02	5.6 ± 1.31
Arg (µM)	155.83 ± 50.4	176.33 ± 48.26	183.92 ± 37.83	155.35 ± 46.71
Arg/ADMA	24.47 ± 4.16	41.84 ± 6.73 *	26.21 ± 4.05	21.11 ± 3.75
ADMA/SDMA	1.23 ± 0.23	1.42 ± 0.37	1.26 ± 0.27	1.45 ± 0.33

**Table 2:** ADMA/SDMA/Arg concentrations in cell culture medium following treatment with ammonia and effect of silencing *Slc7a6* gene silencing. Results are mean ± SD; *n* = 4; * *p* < 0.05 vs. control.

Extracellular Concentration	Control	Ammonia	Mock Control	Ammonia + y^+^LAT2 siRNA
ADMA (µM)	0.365 ± 0.03	0.410 ± 0.04 *	0.358 ± 0.03	0.368 ± 0.05
SDMA (µM)	0.864 ± 0.24	1.378 ± 0.16 *	1.04 ± 0.09	1.189 ± 0.12
Arg (mM)	0.306 ± 0.12	0.312 ± 0.05	0.309 ± 0.04	0.301 ± 0.09
Arg/ADMA	864.24 ± 18.34	756.59 ± 9.66 *	872.33 ± 14.9	813.71 ± 12.28
ADMA/SDMA	0.44 ± 0.05	0.29 ± 0.06	0.36 ± 0.1	0.3 ± 0.04

**Table 3:** The ADMA/SDMA/Arg concentration in (RBE-4) cells. Results are mean ± SD; *n* = 4; * *p* < 0.05 vs. control.

Intracellular Concentration	RBE-4	
	Control	Ammonia
ADMA (µM)	14.27 ± 2.23	8.29 ± 0.84 *
SDMA (µM)	4.29 ± 0.53	2.85 ± 0.31 *
Arg (µM)	134.39 ± 19.13	102.5 ± 11.18
Arg/ADMA	11.20 ± 3.05	14.33 ± 3.80
ADMA/SDMA	3.18 ± 0.25	2.97 ± 0.19
